# Investigating the function of [2Fe–2S] cluster N1a, the off-pathway cluster in complex I, by manipulating its reduction potential

**DOI:** 10.1042/BJ20130606

**Published:** 2013-10-24

**Authors:** James A. Birrell, Klaudia Morina, Hannah R. Bridges, Thorsten Friedrich, Judy Hirst

**Affiliations:** *MRC Mitochondrial Biology Unit, Wellcome Trust/MRC Building, Hills Road, Cambridge CB2 0XY, U.K.; †Institut für Biochemie, Albert-Ludwigs-Universität, Albertstrasse 21, 79104 Freiburg, Germany

**Keywords:** electron transport chain, iron–sulfur cluster, mitochondrion, NADH:quinone oxidoreductase (complex I), oxidative stress, superoxide, APAD^+^, 3-acetylpyridine-adenine dinucleotide, CT-β, C-terminal β-strand, DHE, dihydroethidium, FeCN, ferricyanide, HAR, hexa-ammineruthenium III, HRP, horseradish peroxidase, YPD, 1% (w/v) yeast extract/2% (w/v) peptone/2% (w/v) glucose

## Abstract

NADH:quinone oxidoreductase (complex I) couples NADH oxidation and quinone reduction to proton translocation across an energy-transducing membrane. All complexes I contain a flavin to oxidize NADH, seven iron–sulfur clusters to transfer electrons from the flavin to quinone and an eighth cluster (N1a) on the opposite side of the flavin. The role of cluster N1a is unknown, but *Escherichia coli* complex I has an unusually high-potential cluster N1a and its reduced flavin produces H_2_O_2_, not superoxide, suggesting that cluster N1a may affect reactive oxygen species production. In the present study, we combine protein film voltammetry with mutagenesis in overproduced N1a-binding subunits to identify two residues that switch N1a between its high- (*E. coli*, valine and asparagine) and low- (*Bos taurus* and *Yarrowia lipolytica*, proline and methionine) potential forms. The mutations were incorporated into *E. coli* complex I: cluster N1a could no longer be reduced by NADH, but H_2_O_2_ and superoxide production were unaffected. The reverse mutations (that increase the potential by ~0.16 V) were incorporated into *Y. lipolytica* complex I, but N1a was still not reduced by NADH. We conclude that cluster N1a does not affect reactive oxygen species production by the complex I flavin; it is probably required for enzyme assembly or stability.

## Abstract 2

Two residues that determine the potential of cluster N1a in respiratory complex I were identified, and their effects on its flavin-site reactions were determined. Reduction of cluster N1a by NADH does not affect reactive oxygen species production by the flavin.

## INTRODUCTION

**Figure 1:**
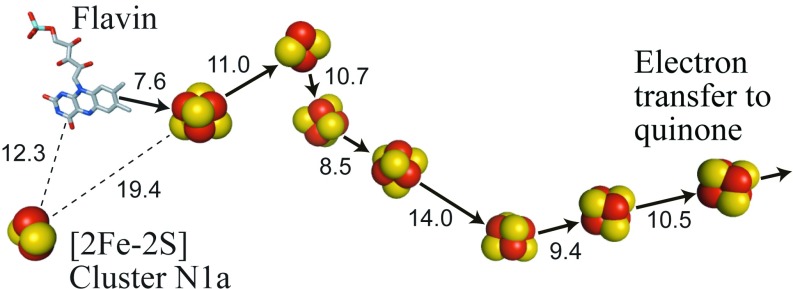
Arrangement of the flavin and Fe–S clusters in complex I The positions of the FMN and eight conserved clusters are shown from the structure of the hydrophilic domain of *T. thermophilus* complex I (PDB code 2FUG) [4]. An additional [4Fe–4S] cluster, found only in *T. thermophilus* and a few other species, is not shown. The distances between the cofactors (between the centres of the two closest ions) are indicated in Ångströms and the pathway from the flavin to the quinone-binding site is shown with solid arrows. The [2Fe–2S] cluster N1a is not part of the pathway; it is on the ‘opposite’ side of the flavin and remote from the other clusters.

Complex I (NADH:quinone oxidoreductase) is an essential respiratory enzyme in many organisms. It forms an entry point to the electron-transport chain, and its dysfunction is linked to numerous human diseases that arise from decreased respiratory capacity and increased oxidative stress [1]. Complex I is a complicated membrane-bound enzyme that catalyses NADH oxidation and quinone reduction, coupled with proton translocation across an energy-transducing membrane [2]. It is composed of a membrane-intrinsic (hydrophobic) domain that transfers the protons and a membrane-extrinsic (hydrophilic) domain that catalyses the redox reaction [3]. NADH is oxidized by hydride transfer to a non-covalently bound FMN at the ‘top’ of the hydrophilic domain. Quinone binds in an extended cavity, with the quinone headgroup bound at the ‘bottom’ of the hydrophilic domain, but elevated above the membrane surface. An extended chain of Fe–S (iron–sulfur) clusters (Figure 1) is essential in transferring electrons from the flavin to the quinone-binding site [2–4].

The structure of the hydrophilic domain of *Thermus thermophilus* complex I revealed the arrangement of the flavin and the Fe–S clusters [4], and a similar arrangement was observed subsequently in complex I from the yeast *Yarrowia lipolytica* [5]. Seven clusters (one [2Fe–2S] and six [4Fe–4S]) link the flavin to the quinone-binding site. An additional [4Fe–4S] cluster is present in just a few species, including *T. thermophilus* and *Escherichia coli*, as a ‘side-branch’ to the chain; it is important for stability of these complexes [6], but not functionally relevant, being more than 20 Å (1 Å=0.1 nm) from any other cofactor. An eighth conserved cluster, a [2Fe–2S] cluster known (historically) as ‘cluster N1a’, is also outside the main cluster chain. It is on the opposite side of the flavin, distant from the other clusters, but close enough to the flavin to exchange electrons rapidly with it (Figure 1). The function of cluster N1a is not known.

Cluster N1a is ligated by the 24 kDa subunit of complex I. We refer in the present paper to the subunits by their names in *Bos taurus*; the homologues of the 24 kDa subunit are NDUFV2 in *Homo sapiens*, NUHM in *Y. lipolytica*, Nqo2 in *T. thermophilus* and NuoE in *E. coli*. The [2Fe–2S] cluster is co-ordinated by a four-cysteine motif at the top of a thioredoxin-like fold in the C-terminal domain of the subunit [4,7]. It is closely associated with the 51 kDa subunit [4,8] (NDUFV1 in *H. sapiens*, NUBM in *Y. lipolytica*, Nqo1 in *T. thermophilus* and NuoF in *E. coli*) that houses the flavin and the NADH-binding site, as well as the first cluster of the chain. The 24 kDa and 51 kDa subunits form a conserved module that is also found in NADPH-dependent hydrogenases [9], and flavoprotein subcomplexes, containing just these two subunits, can be resolved from *B. taurus* complex I using chaotropic agents [8,10], or produced by heterologous expression of the *Paracoccus denitrificans* and *Aquifex aeolicus* genes in *E. coli* [11,12].

The reduced [2Fe–2S]^1+^ cluster N1a exhibits EPR signal N1a, which is characterized by *g* values of 2.00, 1.94 and 1.92, clearly distinct from signal N1b from the [2Fe–2S] cluster in the 75 kDa subunit [13,14]. Signal N1a is observed in NADH-reduced complex I from *E. coli* [15,16], in overproduced 24 kDa subunit homologues [7,17], in the *A. aeolicus* flavoprotein subcomplex [12] and in the *B. taurus* flavoprotein subcomplex reduced by sodium dithionite [10,18]. However, it cannot be observed in NADH-reduced intact complex I from *B. taurus* [14] or in any other intact complex I studied so far (except *E. coli*), including the complexes from *Y. lipolytica* [19], *Pichia pastoris* [20], *P. denitrificans* [21] and *T. thermophilus* [22]. It is likely that cluster N1a remains oxidized in these cases, and the presence of oxidized (EPR-silent) clusters in NADH-reduced complex I from *Y. lipolytica* was confirmed recently using Mössbauer spectroscopy [23].

The reason cluster N1a cannot be reduced by NADH (which sets a potential of ~−0.4 V at pH 7.5) in any species of complex I tested, except for *E. coli*, remains poorly understood. Protein film voltammetry on isolated 24 kDa subunit homologues showed that the *E. coli* cluster has a significantly higher redox potential (−0.28 V) than the *B. taurus*, *P. denitrificans* and *T. thermophilus* clusters (−0.42 to −0.37 V) [7]. However, two further observations indicate that this simple explanation is insufficient. First, signal N1a is observed in the dithionite-reduced flavoprotein subcomplex of *B. taurus* complex I [10], but not in the dithionite-reduced intact enzyme [14]: the properties of the cluster may differ between the isolated subunit, subcomplex and intact enzyme, or supernumerary subunits in the intact enzyme may insulate it from reaction with dithionite. Secondly, signal N1a was not observed in *B. taurus* complex I treated with a very-low-potential (−1 V) Eu(II) reagent [14], supporting the idea that the cluster is insulated from reducing agents in solution and further suggesting that it cannot be reduced by electron transfer from the flavin (otherwise it must have a potential below −1 V). On the other hand, cluster N1a is only 12.3 Å from the flavin (Figure 1), a distance short enough for rapid electron exchange between the two [24].

In complex I from *B. taurus* the NADH-reduced flavin reacts with O_2_ to produce predominantly superoxide, which then dismutates to H_2_O_2_ [25]. The reason the flavin only donates one electron to each O_2_ (rather than donating both electrons to form H_2_O_2_ directly) is not clear. In contrast, the NADH-reduced flavin in *E. coli* complex I does form H_2_O_2_ directly [26], implicating cluster N1a in determining the outcome of O_2_ reduction. It has been suggested that cluster N1a may act as a transient and insulated ‘storage’ site for the second electron, once the first electron has been transferred to the nascent superoxide, and that a similar mechanism may minimize the lifetime of the semi-reduced flavin during catalysis to decrease the overall rate of reactive oxygen species production [27].

In the present study, we aim to define the relationships (in intact complex I) between the reduction potential of cluster N1a, its ability to be reduced by NADH, and the reactivity of the flavin site. First, we identify two mutations that switch the cluster between its ‘high-potential’ (*E. coli*) and ‘low-potential’ (*B. taurus*, *Y. lipolytica* and many other enzymes) forms. Then, we incorporate the mutations into the complexes I from *Y. lipolytica* and *E. coli* to change the *Y. lipolytica* cluster to high-potential and the *E. coli* cluster to low-potential. *Y. lipolytica* is a yeast model system for mitochondrial complex I that is amenable to genetic manipulation of its nuclear-encoded subunits [28]. Finally, we characterize the effects of changing the reduction potential of cluster N1a on the redox reactions catalysed by the flavin site.

## EXPERIMENTAL

### Preparation and characterization of 24 kDa subunit variants

The 24 kDa subunits from *B. taurus* and *E. coli* were overproduced with N-terminal histidine tags using the pMW172 plasmid as described previously [7]; the *B. taurus* protein did not contain the mitochondrion-targeting pre-sequence. Similarly, the mature 24 kDa protein coding sequence from *Y. lipolytica* was amplified from genomic DNA by PCR, and ligated into the pMW172 plasmid with the coding sequence for an N-terminal histidine tag. Site-directed mutagenesis was carried out by PCR using KOD Xtreme Hot Start DNA Polymerase (Novagen) with non-overlapping primers (see Supplementary Table S1 at http://www.biochemj.org/bj/456/bj4560139add.htm for a list of the mutations and primers used). The linear products were 5′-phosphorylated, blunt-end-ligated and transformed into *E. coli* strain XL1-Blue for sequencing, and into *E. coli* strain C41(DE3) [29] for overexpression, with selection by 100 μg·ml^−1^ ampicillin. Sequence-replacement mutants were generated either by ligation of a double-stranded oligonucleotide sequence into a linearized construct lacking the sequence to be replaced or by performing PCR with primers that contained sequence corresponding to the replacement sequence at their 5′-end (see Supplementary Figure S1 at http://www.biochemj.org/bj/456/bj4560139add.htm).

The 24 kDa subunits were overproduced and purified under anaerobic conditions by nickel-affinity chromatography, as described previously [7], and their purity was checked by SDS/PAGE. Cluster reduction potentials were measured anaerobically at 4°C by protein film voltammetry, using a 0.09 cm^2^ pyrolytic graphite edge electrode, in 20 mM Tris/HCl (pH 8) and 0.5 M NaCl, as described previously [7]. Cyclic voltammetry scans at 0.02 V·s^−1^ were initiated at the low-potential limit. The reference electrode was a saturated calomel electrode, and the counter electrode was a platinum wire; all potentials are stated relative to the standard hydrogen electrode.

### Preparation and characterization of *E. coli* and *Y. lipolytica* complexes I with variant 24 kDa subunits

Mutagenesis of the 24 kDa (NuoE) protein in the *E. coli* genome was carried out by QuikChange® (Agilent Technologies), and the mutations were introduced into the pBAD*nuo* expression plasmid by λ-red-mediated recombineering as described previously [30] (see Supplementary Figure S2 and Supplementary Table S2 at http://www.biochemj.org/bj/456/bj4560139add.htm). The *E. coli* cells were grown in LB medium and harvested at stationary phase. Complex I was prepared by isolation of membranes, followed by detergent solubilization, anion-exchange chromatography and nickel-affinity chromatography, as described previously [30].

The pUB26 plasmid, containing a 2.9 kb portion of the *Y. lipolytica* genome that includes the intron-free *nuhm* gene (encoding the 24 kDa subunit homologue from *Y. lipolytica*) and the *tim21* gene, between the ClaI and NheI restriction sites, and the *Y. lipolytica* GB10 *nuhm*Δ strain (that lacks the sequence present in the plasmid in its genomic DNA), were provided by Professor Ulrich Brandt, Zentrum der Biologischen Chemie Fachbereich Medizin, Johann Wolfgang Goethe-Universität, Frankfurt am Main, Germany [28,31]. pUB26 is a shuttle vector which can be genetically manipulated in *E. coli* and used for protein expression in *Y. lipolytica* [28]. It includes ampicillin- and hygromycin B-resistance cassettes, an autonomous replication sequence (ARS68/CEN) and an upstream activating sequence (4× UAS1). Mutagenesis was carried out on the pUB26(*nuhm*) plasmid using the protocol described above for the pMW172 constructs (see Supplementary Table S2). *Y. lipolytica* ΔNUHM GB10 cells were transformed with pUB26(*nuhm*) variants as described previously [32] and selected on YPD [1% (w/v) yeast extract/2% (w/v) peptone/2% (w/v) glucose] agar plates with 100 μg·ml^−1^ hygromycin B at 30°C. Then, single colonies were used to inoculate 2× YPD medium containing 100 μg·ml^−1^ hygromycin B. The cells were grown at 30°C and harvested, and complex I was purified by preparation of mitochondrial membranes, solubilization with detergent and nickel-affinity chromatography, as described previously [20,33,34].

SDS/PAGE analyses were performed with 10–20% acrylamide gels (Invitrogen) and visualized with Coomassie Blue. Blue native PAGE analyses were performed with 3–12% acrylamide gels (Invitrogen) and visualized in the same way. In-gel complex I activities were measured by incubating the gel in 20 mM Tris/HCl (pH 7.5), 0.5 mg·ml^−1^ Nitro Blue Tetrazolium and 120 μM NADH. Protein concentrations were measured using the Pierce BCA assay, and flavin concentrations were analysed fluorimetrically [35]. Flavin-site stability was assessed using a method based on the ‘ThermoFAD’ protocol described previously [36]. An ABI 7900HT real-time PCR machine was used to monitor the fluorescence of free flavin released from complex I. The temperature was held at 30°C for 2 min, and then increased by 1.5°C every 30 s. For MS analyses, ~10 μg of protein samples was reduced and alkylated with *N*-ethylmaleimide, and then digested with trypsin. The peptide digests were analysed by LC–MS/MS using a nano-scale reverse-phase separation column (75 μm×100 mm; Nano-Separations) and an LTQ OrbiTrap XL mass spectrometer (Thermo Fisher). Peptide mass and fragment data were compared with the NCBI (National Center for Biotechnology Information) sequence database using Mascot (Matrix Sciences) [37]. Relative peptide abundances (for the same peptide in different samples) were estimated by comparison of peak volumes.

### EPR spectroscopy

Complex I samples (typically 200 μl of 10 mg·ml^−1^) were reduced under anaerobic conditions with NADH and frozen immediately. EPR spectra were recorded using a Bruker EMX X-band spectrometer with an ER 4119HS high-sensitivity cavity and maintained at a low temperature (4–40 K) by an ESR900 continuous-flow liquid helium cryostat (Oxford Instruments). The parameters used were microwave frequency 9.38–9.39 GHz, modulation frequency 100 kHz, modulation amplitude 1 mT, time constant 81.92 ms and conversion time 20.48 ms, with the microwave power and temperature specified in the Figure legends.

### Kinetic assays

The rates of the NADH:FeCN (ferricyanide), NADH:HAR (hexa-ammineruthenium III) and NADH:APAD^+^ (3-acetylpyridine-adenine dinucleotide) oxidoreduction reactions were measured as described previously [38]. H_2_O_2_ was detected by the HRP (horseradish peroxidase; 10 units·ml^−1^)-mediated conversion of 10 μM Amplex Red into resorufin (557–620 nm, ϵ=51.6 mM^−1^·cm^−1^) [25] with background rates in the presence of catalase (bovine liver; 1000 units·ml^−1^) subtracted. Superoxide was detected by the oxidation of DHE (dihydroethidium; 50 μM) to ethidium, followed by its intercalation into DNA (salmon sperm; 50 μg·ml^−1^), with background rates in the presence of superoxide dismutase and the absence of complex I subtracted [26]. The response was monitored by fluorescence (excitation at 396 nm and emission at 590 nm) and calibrated using the known rates of superoxide production by *B. taurus* complex I. All assays were performed at 32°C in a 20 mM Tris/HCl buffer (pH 7.5).

## RESULTS AND DISCUSSION

### Reduction potentials of the [2Fe–2S] clusters in the 24 kDa homologues are determined by hydrogen-bonding

**Figure 2:**
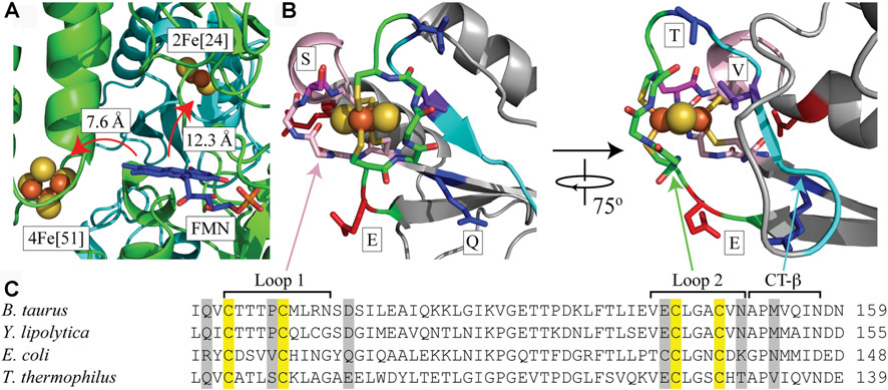
The environment of the [2Fe–2S] cluster in the 24 kDa subunit of complex I (**A**) The region around the FMN in the structure of the hydrophilic domain of complex I from *T. thermophilus* (PDB code 2FUG) [4]. The positions of the two flavin-adjacent clusters, a [4Fe–4S] cluster in the 51 kDa subunit and the [2Fe–2S] cluster in the 24 kDa subunit, are shown with respect to the FMN. (**B**) The structure of the 24 kDa (Nqo2) subunit from two perspectives highlighting the [2Fe–2S] cluster, its first and second co-ordination loops (pink and green respectively), and the C-terminal β-strand (CT-β, in cyan). The positions of amino acids mutated in the present study are indicated; the two negatively charged amino acids (changed to neutral) are in red (E), the two neutral amino acids (changed to positive) are in blue (Q and T), and the two hydrogen-bonding residues are in dark pink (S) and purple (V). (**C**) Alignment of the sequences of the 24 kDa subunit (*B. taurus*, UniProt code P04394), NUHM (*Y. lipolytica*, UniProt code Q9UUT9), NuoE (*E. coli*, UniProt code P0AFD1) and Nqo2 (*T. thermophilus*, UniProt code Q56221), indicating important features. The numbers at the end of the sequence indicate the position of the last amino acid shown with respect to the start of the mature protein.

**Figure 3:**
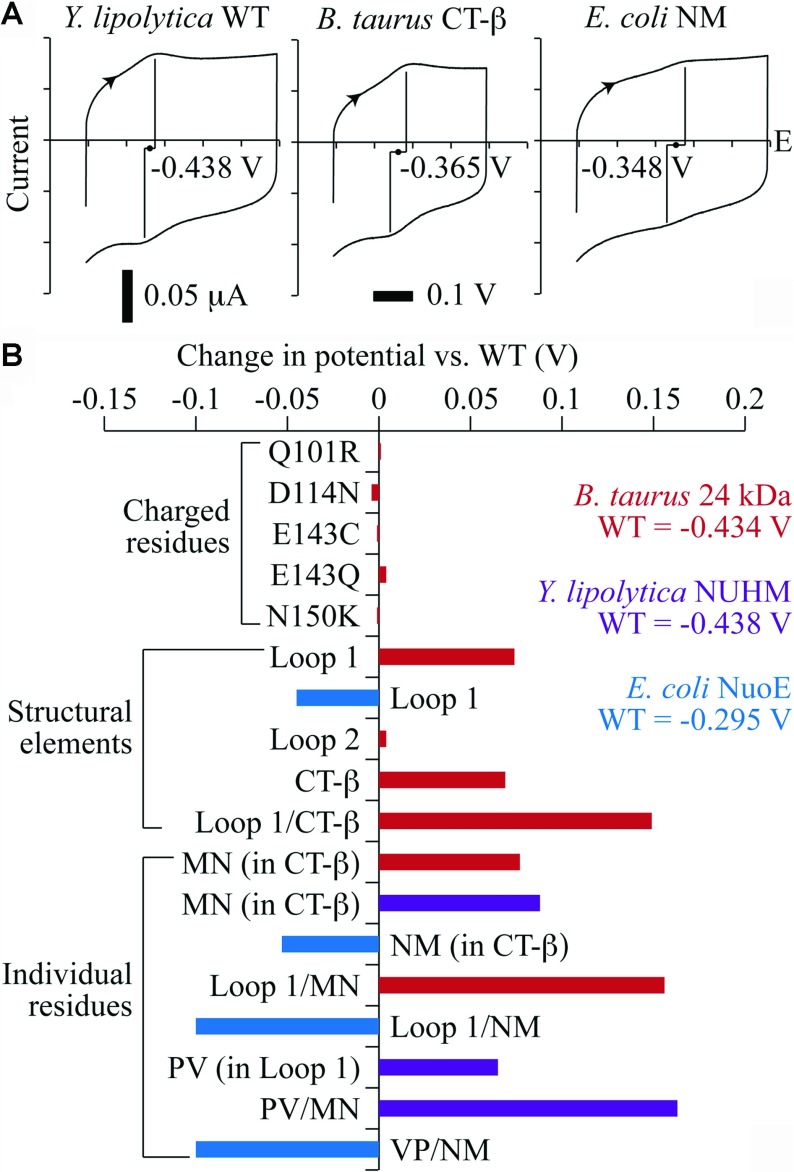
Reduction potentials of the [2Fe–2S] clusters in the overproduced 24 kDa subunit variants (**A**) Three examples of protein film voltammetry measurements of the reduction potentials of the [2Fe–2S] clusters in the variant proteins. The three cyclic voltammograms are all shown on the same scale; the reduction potentials are the average of the peak positions in the two scan directions. The proteins were adsorbed on a pyrolytic graphite edge electrode and the potential scanned at 0.02 V·s^−1^ from low to high potential and back at 4°C in a buffer containing 20 mM Tris/HCl (pH 8) and 0.5 M NaCl. Potentials are reported relative to the standard hydrogen electrode. (**B**) Reduction potentials of subunit variants, relative to their wild-type values, measured as in (**A**), with values from the *B. taurus* 24 kDa subunit in red, *Y. lipolytica* NUHM in purple and *E. coli* NuoE in blue.

Our first objective was to identify the residues that determine whether N1a is a ‘high-potential’ cluster, as in *E. coli*, or a ‘low-potential’ cluster, as in *B. taurus* and *Y. lipolytica* (Figure 2). The potentials of the clusters in the overproduced subunits from *B. taurus*, *P. denitrificans* and *T. thermophilus* (low-potential) and *E. coli* (high-potential) were measured previously using protein film voltammetry [7,39]. In the present study, we focus on the proteins from *B. taurus* and *E. coli*, and also *Y. lipolytica*, a yeast model system for mitochondrial complex I that enables mutagenesis in the intact enzyme [40]. The 24 kDa subunit from *Y. lipolytica* was overproduced in *E. coli*, and protein film voltammetry was used to show that (as expected) it has a low potential. To test the effects of specific residues, mutations were generated in the *B. taurus*, *Y. lipolytica* and *E. coli* subunits and their cluster potentials were measured. Figure 3 shows a representative set of voltammograms and summarizes the results.

First, changes in the electrostatic environment of the cluster were considered. Sequence alignments were used to identify 18 residues that are negatively charged in the low-potential proteins and neutral in the high-potential protein, or neutral in the low-potential proteins and positively charged in the high-potential protein. Four of them are close to the cluster (Figure 2) [4] and so were selected for mutagenesis. However, changing these residues in the *B. taurus* subunit (Gln^101^, Asp^114^, Glu^143^ and Asn^150^) to their equivalent residues from the *E. coli* sequence (or, for the E143Q mutation, removing the charge) did not change the cluster potential significantly (Figure 3B). Therefore these residues are not important for defining the cluster N1a potential.

Secondly, stretches of sequence corresponding to secondary structural elements were identified for exchange between the high- and low-potential proteins. Inspection of the structure [4] revealed the cluster-co-ordinating loops (Loop 1 and Loop 2), and a β-strand C-terminal to one of these loops (CT-β), as candidates (Figure 2). Swapping Loop 1 and CT-β from the high-potential *E. coli* sequence into the low-potential *B. taurus* protein increased the cluster reduction potential by 74 mV and 69 mV respectively, and the combined replacement (Loop 1/CT-β) increased it by 139 mV; to a value very close to that of the high-potential protein itself (Figure 3B). Swapping Loop 2 had no effect.

CT-β of the *E. coli* protein contains an asparagine residue (Asn^142^), which is replaced by a methionine residue in the *B. taurus* and *Y. lipolytica* proteins (Figure 2). The *B. taurus* M153N and *Y. lipolytica* M149N mutations increased the cluster potential by 77 mV and 88 mV respectively, and the *E. coli* N142M mutation decreased it by 53 mV (Figure 3B). The primary amide of *E. coli* Asn^142^ probably forms a hydrogen bond to one of the μ_2_-sulfides of the [2Fe–2S] cluster, delocalizing the electron density and increasing the potential. The effects of hydrogen-bonding on cluster potential have been well documented in other Fe–S proteins, including the Rieske protein [41] and rubredoxins [42]. Combining the M153N mutation with the *E. coli* Loop 1 replacement (Loop 1/MN) in the *B. taurus* protein increased the cluster potential by 156 mV. Combining the N142M mutation with the *B. taurus* Loop 1 replacement (Loop 1/NM) in the *E. coli* protein decreased the cluster potential by 100 mV.

Loop 1 in *E. coli* contains a valine residue (Val^96^) that is replaced by proline in *B. taurus* (Pro^107^) and *Y. lipolytica* (Pro^103^) (Figure 2). The P103V mutation and combined P103V and M149N (PV/MN) mutations in *Y. lipolytica* increased the cluster potential by 65 mV and 163 mV respectively. The combined V96P and N142M mutations in *E. coli* (VP/NM) decreased the cluster potential by 100 mV (Figure 3B). The valine-to-proline substitution replaces a backbone secondary amide, which is close to the cluster and so may form a hydrogen bond to it, with a tertiary amide. It may also affect the protein conformation; replacing a proline residue adjacent to the [3Fe–4S] cluster in *Azotobacter vinelandii* ferredoxin I caused an additional water molecule to enter the structure, increasing the cluster potential by a similar amount to observed in the present study [43].

To conclude, cluster N1a can be switched between its high- and low-potential forms in the isolated 24 kDa subunits by exchanging two residues: a residue in the first clusterco-ordinating loop (Pro^107^ in *B. taurus* and Pro^103^ in *Y. lipolytica*, low-potential, or Val^96^ in *E. coli*, high-potential) and a residue in the β-strand C-terminal to the second cluster-co-ordinating loop (Met^153^ in *B. taurus* and Met^149^ in *Y. lipolytica*, low-potential, or Asn^142^ in *E. coli*, high potential). Exchanging both residues increases the potential in the *B. taurus* and *Y. lipolytica* proteins by ~160 mV and decreases the potential in the *E. coli* protein by 100 mV. The wild-type cluster potentials differ by 140 mV.

### EPR demonstrates that the V96P and N142M mutations in *E. coli* complex I decrease the cluster N1a reduction potential

**Figure 4:**
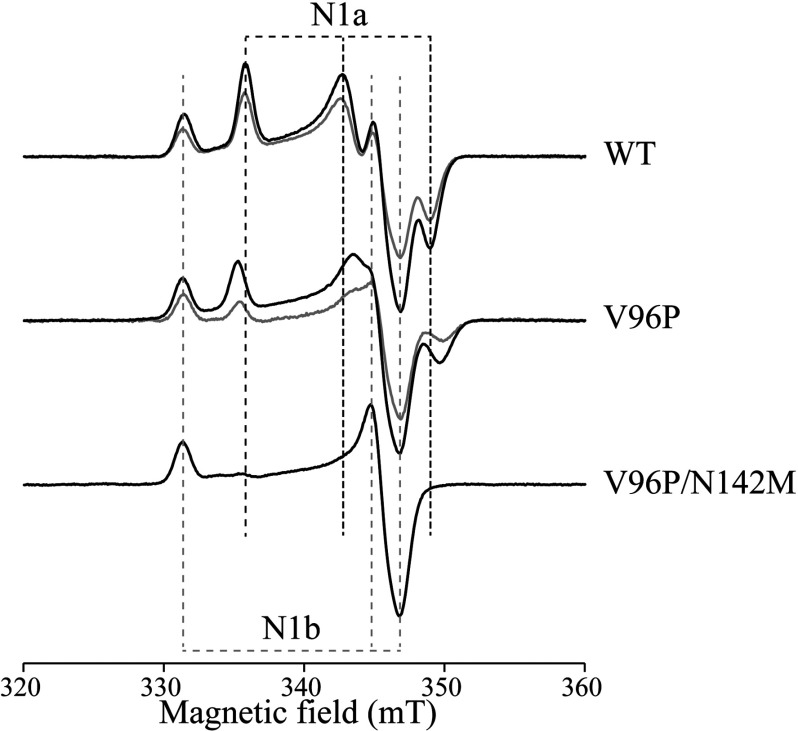
EPR spectra of variants of complex I from *E. coli* The spectrum of the NADH-reduced wild-type (WT) enzyme is compared with spectra from the NuoE V96P and V96P/N142M variants. Black spectra are from enzymes reduced with 5 mM NADH, under anaerobic conditions, to approximately −0.37 V (at pH 6.5). Grey spectra are from enzymes reduced with 1 mM NADH and 1 mM NAD^+^, to set the potential to approximately −0.31 V (at pH 6.5). The spectra all show a clear N1b signal, from the [2Fe–2S] cluster in the 75 kDa (NuoG) subunit; the N1a signal from the [2Fe–2S] cluster in the 24 kDa (NuoE) subunit is clearly visible in the WT and V96P spectra, but not in the V96P/N142M spectrum. The *g*_z_-signal from N1a is shifted in V96P. Spectra were recorded at 40 K and 1 mW microwave power, and have been normalized to the protein concentrations. The other parameters were: microwave frequency 9.38–9.39 GHz, modulation frequency 100 kHz, modulation amplitude 1 mT, time constant 81.92 ms, and conversion time 20.48 ms. Protein samples were in a buffer containing 50 mM Mes/OH (pH 6.5), 50 mM NaCl, 0.1% *n*-dodecyl-β-D-maltoside and 10% glycerol.

The V96P and N142M mutations in the 24 kDa (NuoE) subunit were incorporated, sequentially, into *E. coli* complex I using λ-red-mediated recombineering [30] (Supplementary Figure S2) to provide the V96P and V96P/N142M strains. Complex I was purified from each strain [30], and the wild-type, V96P and V96P/N142M complexes were reduced by NADH (to approximately −0.37 V at pH 6.5) and characterized by EPR (Figure 4, black traces).

In wild-type *E. coli* complex I, the N1a cluster potential is −0.25 V [15], and a similar potential (−0.295 V) was measured in the present study using the isolated subunit. Consistent with these values, cluster N1a is reduced by NADH in the wild-type enzyme (Figure 4). In the NADH-reduced V96P variant the intensity of signal N1a is decreased by ~40% relative to the wild-type (the spectra in Figure 4 have been normalized to protein concentration so the N1b signal, from a different [2Fe–2S] cluster, does not change). Therefore the V96P cluster is only partially reduced by NADH. For wild-type *E. coli* complex I, increasing the NADH potential to −0.31 V (by using equimolar amounts of NADH and NAD^+^ at pH 6.5) caused both the N1a and N1b signal intensities to decrease by ~30% (Figure 4, grey traces), but for V96P the N1a intensity is decreased much more (by ~70%). These observations confirm that the N1a potential has shifted negatively in V96P. In addition, the N1a *g*_z_ and *g*_x_ features have shifted slightly (from 1.996 to 1.999 and 1.920 to 1.916 respectively), consistent with an alteration of the cluster environment.

Signal N1a is absent from the spectrum of the NADH-reduced V96P/N142M variant (Figure 4), indicating that cluster N1a cannot be reduced by NADH because its potential has shifted more negatively. For the cluster to be <10% reduced at −0.37 V it must have a reduction potential below −0.43 V. Thus the two mutations have shifted the potential more in the intact *E. coli* enzyme than in the isolated *E. coli* subunit, but by an amount consistent with the double mutations in the isolated *B. taurus* and *Y. lipolytica* subunits. The difference is probably due to small changes to the cluster environment upon its incorporation into the complex. The alternative explanation, that the cluster is not present in the V96P/N142M variant, is highly unlikely to be correct. When we intentionally disrupted cluster N1a by mutating one of its cysteine ligands to either an alanine or serine residue, complex I activities in the resulting membrane preparations were very low, and any complex present was too unstable to be isolated (K. Dörner, M. Vranas, J. Hoeser, I. Straub, D. Thiel and T. Friedrich, unpublished work). In contrast, all of the proteins described in the present study were purified normally using standard protocols. To quantify complex I activities in the *E. coli* membranes, we used a strain that lacks both alternative NADH dehydrogenases transformed with the complex I variants. Comparison of the rates from the wild-type and V96P/N142M variants shows that the V96P/N142M membranes exhibit ~80% of the wild-type activity (see Supplementary Table S3 at http://www.biochemj.org/bj/456/bj4560139add.htm). SDS/PAGE analyses of the purified enzymes showed the expected band pattern (see Supplementary Figure S3 at http://www.biochemj.org/bj/456/bj4560139add.htm), with the 24 kDa subunit visible, and the presence of both the subunits and the mutations were confirmed by MS. The wild-type and V96P/N142M variants were estimated to contain 1.24±0.22 and 1.26±0.17 flavins per complex respectively, and EPR spectra recorded at low temperatures, to probe the complex I [4Fe–4S] clusters, revealed no differences (except for signal N1a; see Supplementary Figure S4 at http://www.biochemj.org/bj/456/bj4560139add.htm). A small decrease in the stability of the flavin site in V96P/N142M, relative to the wild-type enzyme was observed (see Supplementary Figure S5 at http://www.biochemj.org/bj/456/bj4560139add.htm), consistent with the small changes in the reactivity of the flavin site discussed below. Importantly, these minor changes are very different from the global effects on stability and activity that result when cluster N1a is not present.

To conclude, the potential of cluster N1a in *E. coli* complex I can be switched from its high-potential NADH-reducible form to a low-potential form that cannot be reduced by NADH by incorporating the V96P and N142M mutations into the 24 kDa subunit. This provides a ‘mitochondrial-type’ enzyme for comparison with the wild-type enzyme, in order to elucidate the functional effects of the N1a cluster potential on the flavin site (see below).

### The P103V and M149N mutations do not enable cluster N1a reduction in *Y. lipolytica* complex I

The P103V and M149N mutations were introduced, individually and in combination, into the 24 kDa subunit of *Y. lipolytica* complex I by using an *nuhm*-knockout strain (*Y. lipolytica* GB10 *nuhm*Δ) [28]. GB10 *nuhm*Δ expresses an alternative NADH dehydrogenase redirected to the matrix side of the inner mitochondrial membrane [44] and a histidine-tagged form of NUGM (the 30 kDa subunit) [33]. It is incapable of correct complex I assembly (but viable due to the alternative dehydrogenase) unless complemented by the expression of NUHM (the 24 kDa subunit) from the replicative pUB26 plasmid [28].

**Figure 5:**
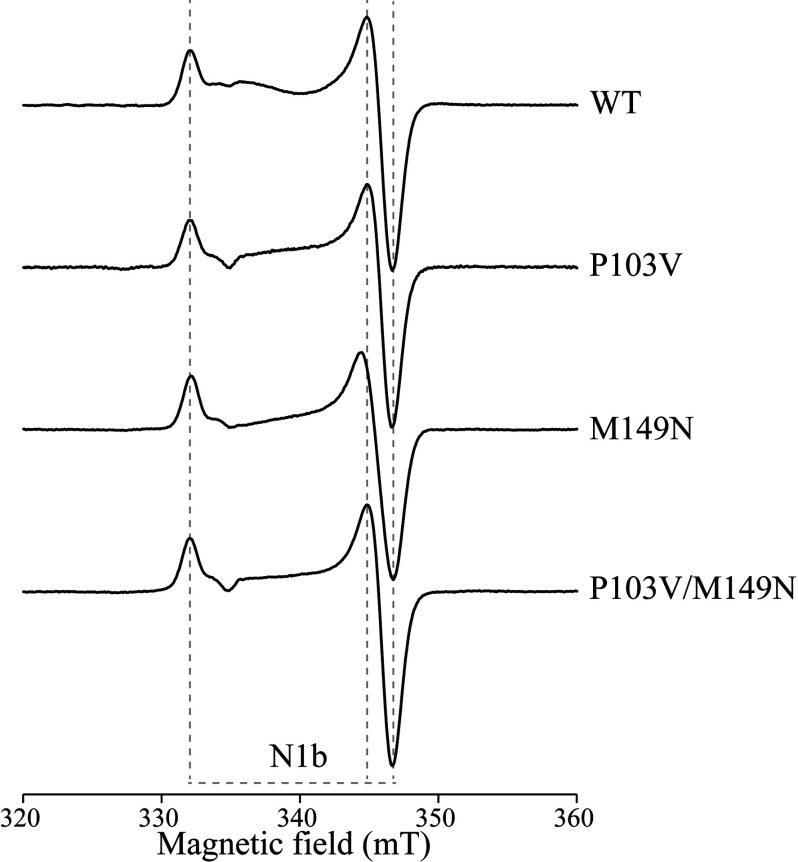
EPR spectra of variants of complex I from *Y. lipolytica* The spectrum of the NADH-reduced wild-type (WT) enzyme is compared with spectra from the P103V, M149N and P103V/M149N NUHM variants. The spectra all show a clear N1b signal from the [2Fe–2S] cluster in the 75 kDa (NUAM) subunit; none of them shows the N1a signal from the [2Fe–2S] cluster in the 24 kDa (NUHM) subunit. The enzymes were reduced with 5 mM NADH, under anaerobic conditions; spectra were recorded at 40 K and 1 mW microwave power, and have been normalized to the protein concentrations. The other parameters were: microwave frequency 9.38–9.39 GHz, modulation frequency 100 kHz, modulation amplitude 1 mT, time constant 81.92 ms, and conversion time 20.48 ms. Protein samples were in a buffer containing 20 mM Mops/OH (pH 7.5), 150 mM NaCl, 0.02% *n*-dodecyl-β-D-maltoside and 10% glycerol.

Complex I was purified from the wild-type, P103V, M149N and P103V/M149N strains, and then samples were reduced by NADH and investigated by EPR. However, none of them displayed the N1a signal expected from the ‘high-potential’-reduced cluster N1a (Figure 5). Samples were also reduced with sodium dithionite, but their EPR spectra were identical with those of the NADH-reduced complexes. These results are surprising, because they indicate that the cluster potential is still too low, even in the P103V/M149N variant, for the cluster to be reduced. As discussed for *E. coli* complex I above, the alternative explanation, that the mutations prevent cluster incorporation, is very unlikely. The wild-type and variant strains grew at similar rates, so the mutations do not confer a detrimental growth phenotype. Blue native PAGE analyses confirmed that similar amounts of complex I were produced in wild-type and P103V/M149N membranes (as well as in the parent GB10 strain in which the 24 kDa subunit is expressed from genomic DNA) and in-gel assays confirmed that their flavin-site activities were similar (see Supplementary Figure S6 at http://www.biochemj.org/bj/456/bj4560139add.htm). All of the variants were purified using the standard protocol, and the band patterns observed in SDS/PAGE analyses were normal (see Supplementary Figure S7 at http://www.biochemj.org/bj/456/bj4560139add.htm), with the band from the 24 kDa subunit clearly visible. The relative abundances of peptides from the 24 kDa and 51 kDa (NUBM) subunits, compared using MS, were similar in the wild-type and P103V/M149N variants, and MS also confirmed the presence of the mutations. The wild-type and P103V/M149N variants were estimated to contain 1.20±0.19 and 1.13±0.10 flavins per complex respectively, and the thermal stabilities of the flavin sites were essentially identical (see Supplementary Figure S8 at http://www.biochemj.org/bj/456/bj4560139add.htm). EPR spectra recorded at lower temperatures, to investigate the [4Fe–4S] cluster cohort, confirmed that all of the expected signals were present in each variant, and revealed no differences between them (see Supplementary Figure S9 at http://www.biochemj.org/bj/456/bj4560139add.htm). Each variant also exhibited substantial flavin-site catalytic activities (see Supplementary Table S4 at http://www.biochemj.org/bj/456/bj4560139add.htm). In striking contrast with these results, mutagenesis of the cysteine residues that co-ordinate the N1a cluster in *Y. lipolytica*, or mutation of a nearby methionine residue to lysine (structurally hindering cluster incorporation), prevented complex I assembly altogether [31]; similarly, mutations of the cluster ligands in *Neurospora crassa* showed that incorporation of cluster N1a is required for protein assembly [45].

It is possible that the N1a cluster potential may be much lower in intact mitochondrial complex I than in the isolated subunit, so that the higher cluster potentials in the variants are still too low for reduction by NADH or dithionite. In addition, reduction of the cluster ensemble (which differs between the *E. coli* and mitochondrial enzymes [13]) may ‘push’ the N1a potential progressively more negative. Consistent with this thermodynamic explanation, only approximately half the [4Fe–4S] clusters are reduced by NADH in *Y. lipolytica* complex I [23], and EPR signal N1a could not be observed in *B. taurus* complex I even when it was reduced to −1 V [14]. Alternatively, electron transfer from the reduced flavin to cluster N1a may be very slow kinetically. Although the distance between the cluster and the flavin is short enough for facile electron transfer [4,5], it may be that an electron, partitioned between the flavin and cluster N1a in mitochondrial complex I, always strongly favours the flavin. However, reduction of the cluster should still be achieved eventually, and this has not been observed.

### Altering the reduction potential of the [2Fe–2S] cluster in *E. coli* complex I does not affect the stoichiometry of reactive oxygen species production

**Figure 6:**
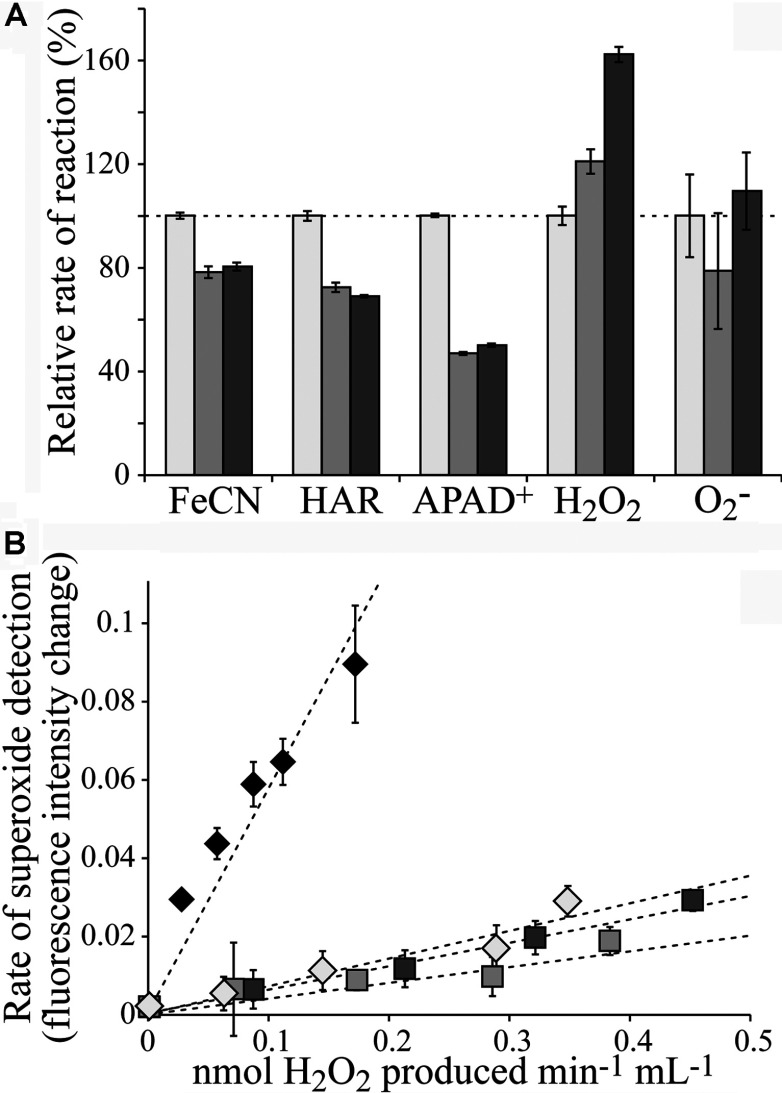
Flavin-site reactions catalysed by the 24 kDa subunit variants of complex I from *E. coli* (**A**) The rates of NADH oxidation (30 μM NADH), coupled with the reduction of FeCN (1 mM), HAR (0.5 mM) and APAD^+^ (0.5 mM), and coupled with O_2_ reduction detected as H_2_O_2_ or O_2_^−^. H_2_O_2_ was detected using the HRP–Amplex Red assay (10 μM Amplex Red and 2 units·ml^−1^ HRP), and O_2_^−^ using DHE (50 μM DHE and 50 μg·ml^−1^ salmon sperm DNA). The rates for V96P (medium grey) and V96P/N142M (dark grey) are expressed relative to the wild-type rates (light grey). All assays were performed in a buffer containing 20 mM Tris/HCl (pH 7.5) and at 32°C. (**B**) Comparison of the ratio of H_2_O_2_ to O_2_^−^ production. The total rates of H_2_O_2_ formation (from the dismutation of O_2_^−^ and from H_2_O_2_ formed directly) were measured, over a range of complex I concentrations, and are plotted on the *x*-axis. They are compared with the rates of fluorescence intensity change of DHE, representing the relative rates of O_2_^−^ production, by comparison with data from bovine complex I (black diamonds); bovine complex I produces predominantly O_2_^−^ [25]. The wild-type enzyme (light grey diamonds) and both the variants (medium and dark grey squares) produce predominantly H_2_O_2_. Assays were in 30 μM NADH, in a buffer containing 20 mM Tris/HCl (pH 7.5), at 32°C.

Figure 6(A) shows that the rates of several flavin-site reactions are moderately decreased in the *E. coli* complex I variants relative to the wild-type. NADH:APAD^+^ oxidoreduction (a transhydrogenase reaction [46]) is most affected. The NADH:FeCN [38] and NADH:HAR [47] reactions are also decreased, but the rate of NADH-linked H_2_O_2_ production (that represents the total rate of NADH:O_2_ oxidoreduction) is increased. These observations suggest a small perturbation of the flavin site that weakens nucleotide binding, especially when the flavin is reduced; the NADH:O_2_ reaction is strongly inhibited by NADH binding to the reduced flavin [38]. Measuring the rate of superoxide production is problematic for *E. coli* complex I, due to high background rates between the enzyme and acetylated cytochrome *c*, the method of choice for *B. taurus* complex I [25]. Therefore superoxide production was measured using DHE [26] (Figure 6B). Figure 6(B) shows that the superoxide/H_2_O_2_ ratio is not affected by the mutations, so switching from a fully reduced to a fully oxidized cluster N1a does not turn *E. coli* complex I into a superoxide-producing enzyme, and the redox status and reduction potential of [2Fe–2S] cluster N1a in the 24 kDa subunit does not determine the identity of reactive oxygen species produced. Therefore our results do not support cluster N1a as a transient store of an electron from the semi-reduced flavin, either to minimize reactive oxygen species production during turnover [4] or to preclude the direct production of H_2_O_2_ [26]. Thus it is unlikely that cluster N1a has a specific functional role in complex I. Instead, because mutations of the cluster ligands in both *E. coli* and *Y. lipolytica* preclude formation of a stable and functional enzyme, it is most likely that the correct incorporation of cluster N1a is required for enzyme assembly and stability.

It remains unclear why the fully reduced flavin in mitochondrial complex I produces superoxide when the same cofactor in *E. coli* produces H_2_O_2_ [25,26]. Reduction of O_2_ to H_2_O_2_ is a sequential process; in mitochondrial complex I reduction of the nascent superoxide does not compete effectively with its escape from the active site. Do subtle structural features of the *E. coli* active site retain the nascent superoxide, increasing the chance of superoxide reduction? Alternatively, superoxide reduction may be hindered in the mitochondrial enzyme by unknown features that prevent protonation or intersystem crossing (the spin change required during the two-electron reduction of ^3^O_2_) or the electron from the semi-flavin may be rapidly redistributed to the main cluster chain, as proposed previously for fumarate reductase [48] and xanthine dehydrogenase [49].

## Online data

Supplementary data
