# Supplementary material for: Investigating the function of [2Fe–2S] cluster N1a, the off-pathway cluster in complex I, by manipulating its reduction potential
Source: Biochem J. 2013 Oct 24;456(Pt 1):139–46. doi: 10.1042/BJ20130606 (PMC3898324; doi:10.1042/BJ20130606)
Supplement: Supplementary data [file bj4560139add.pdf]

BJ Energy

Biochem. J. (2013) **456**, 139–146 (Printed in Great Britain) doi:10.1042/BJ20130606

online data

SUPPLEMENTARY ONLINE DATA

Investigating the function of [2Fe–2S] cluster N1a, the off-pathway cluster in complex I, by manipulating its reduction potential

James A. BIRRELL*, Klaudia MORINA†, Hannah R. BRIDGES*, Thorsten FRIEDRICH† and Judy HIRST*[1]

*MRC Mitochondrial Biology Unit, Wellcome Trust/MRC Building, Hills Road, Cambridge CB2 0XY, U.K., and †Institut für Biochemie, Albert-Ludwigs-Universität, Albertstrasse 21, 79104 Freiburg, Germany

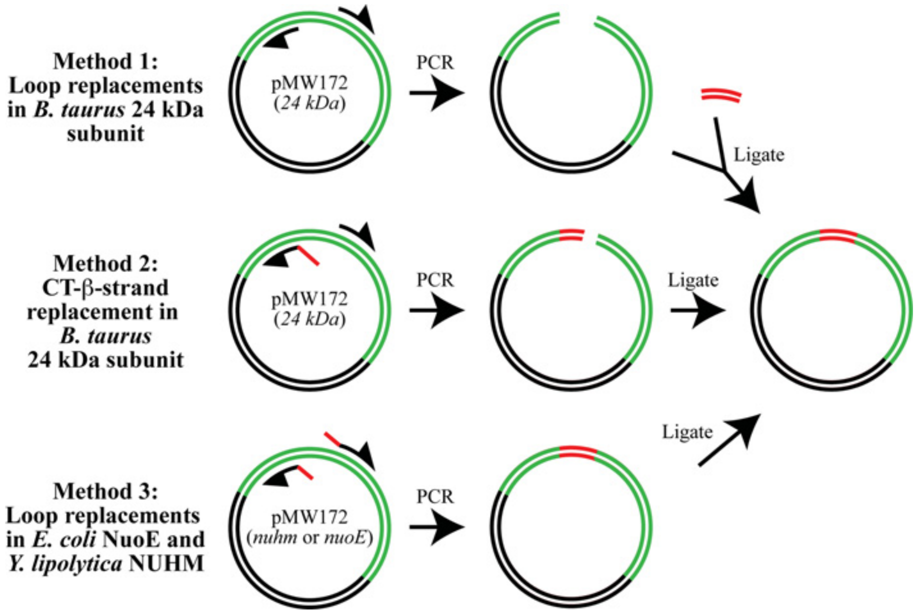

Figure S1 Strategies for generating loop and CT-β strand replacements in the 24 kDa subunit plasmids

The double circle (black and green) represents the pMW172 plasmid (black) containing the 24 kDa subunit protein-coding sequences (green). Black curved half-arrows represent the PCR primers used to generate linear double-stranded DNA fragments. The red lines represent the replacement sequence. The 24 kDa protein variants, with portions of the sequence replaced with homologous sequence from another species, were generated using three methods. (1) PCR was used to amplify a linear form of the parent plasmid without the region to be replaced (primers 13/14 and 19/20, Table S1), and then blunt-end ligation was used to insert the replacement sequence (primers 15/16 and 21/22, Table S1). (2) PCR was used to amplify a linear form of the parent plasmid that contained the replacement sequence (primers 25/26, Table S1), and then blunt-end ligation was used to circularize the plasmid. (3) PCR using primers (17/18 and 23/24, Table S1) that both contained half of the replacement sequence was used to amplify a linear form of the parent plasmid, and then blunt-end ligation was used to circularize the plasmid. Method 1 was used in earlier experiments, but produced false positives due to sequence insertion in the wrong direction. Methods 2 and 3 were used in later experiments; both methods were effective, but PCR was more successful with method 3 because the primers required are shorter and contain less mismatched sequence.

[1] To whom correspondence should be addressed (email jh@mrc-mbu.cam.ac.uk).

© 2013 The Author(s)
The author(s) has paid for this article to be freely available under the terms of the Creative Commons Attribution Licence (CC-BY) (http://creativecommons.org/licenses/by/3.0/) which permits unrestricted use, distribution and reproduction in any medium, provided the original work is properly cited.

Biochemical Journal www.biochemj.org

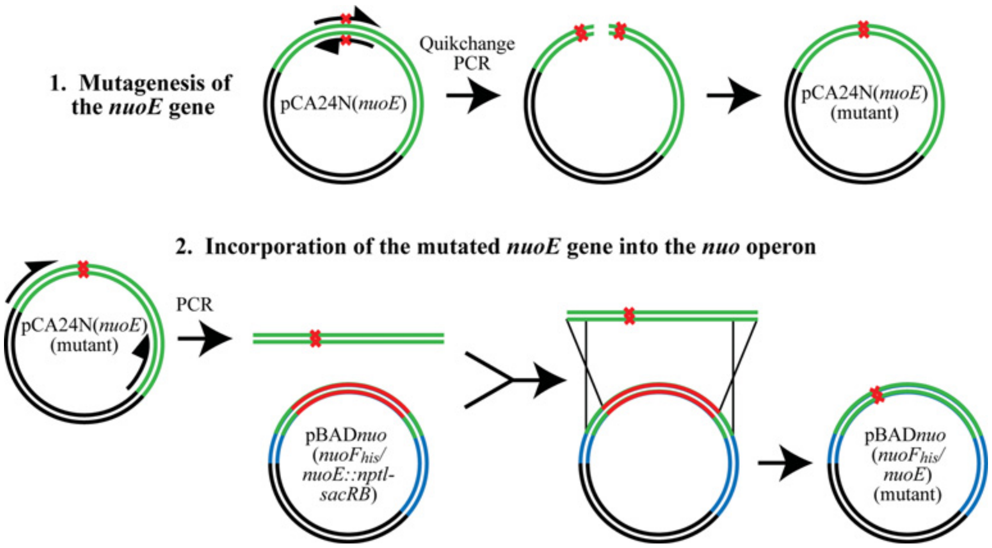

Figure S2 λ-Red-mediated recombineering strategy used to generate mutations in the 24 kDa subunit homologue (NuoE) in *E. coli* complex I

The green and black double circle [pCA24N(*nuoE*)] represents the plasmid containing the *nuoE* gene (green). The multicoloured double circle [pBAD*nuo*(*nuoF*$_{his}$*/nuoE::nptI-sacRB*)] represents the plasmid containing the whole *nuo* operon (blue), including the *nuoE* gene (green) which has been disrupted with the *nptI-sacRB* cartridge (red) from pUM24. (1) pCA24N(*nuoE*) was used for mutagenesis of the *nuoE* gene by QuikChange® (primers 37/38 and 39/40, Table S2) and the products were used to co-transform *E. coli* DH5α; the plasmid was amplified and purified. (2) PCR was used to amplify a linear fragment that included the mutated *nuoE* sequence (primers 41/42, Table S2), and this was used, in combination with pBAD*nuo*(*nuoF*$_{his}$*/nuoE*) to transform *E. coli* DH5$\alpha\Delta$*nuo*/pKD46. Subsequently, site-specific recombination restored the complete *nuo* operon.

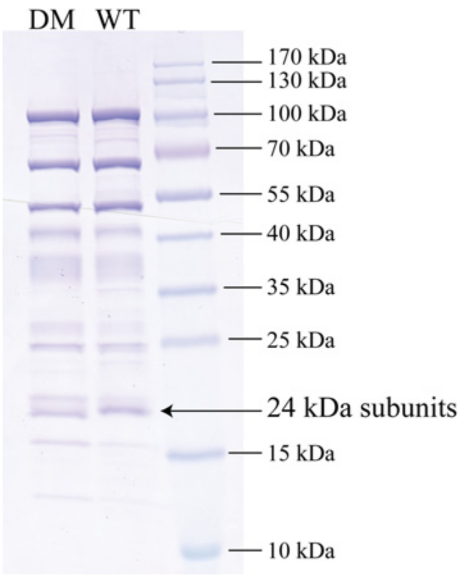

Figure S3 SDS/PAGE analyses of the wild-type and V96P/N142M (double mutant) variants of purified *E. coli* complex I

The sizes of a set of molecular-mass markers, and the positions of the 24 kDa subunits, are indicated. WT, wild-type; DM, double mutant (V96P/N142M). A total of 5 μg of protein was loaded per lane.

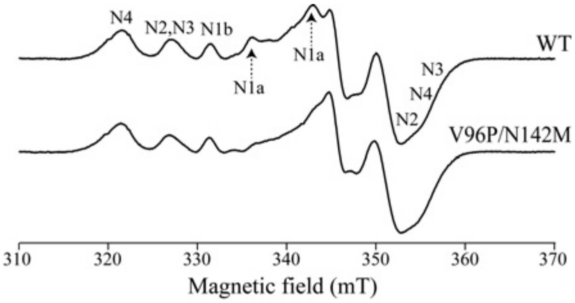

Figure S4 EPR spectra of the [4Fe–4S] clusters in the wild-type and V96P/N142M variants of *E. coli* complex I

Spectra were recorded at 12 K to focus on the complement of [4Fe–4S] clusters present, using 1 mW microwave power (see the legend to Figure 4 in the main paper for other experimental details). Key features from the clusters observed are marked (the [4Fe–4S] clusters are N2, N3 and N4). With the exception of the expected absence of signal N1a (present at low intensity at 12 K), the spectra of the wild-type (WT) and V96P/N142M variants are essentially indistinguishable.

© 2013 The Author(s)
The author(s) has paid for this article to be freely available under the terms of the Creative Commons Attribution Licence (CC-BY) (http://creativecommons.org/licenses/by/3.0/) which permits unrestricted use, distribution and reproduction in any medium, provided the original work is properly cited.

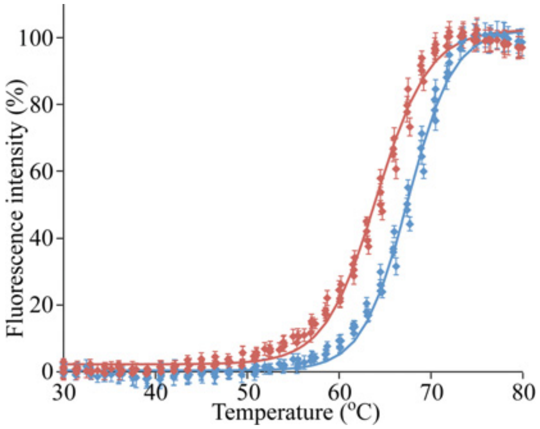

Figure S5 Thermal stability of the flavin site in the WT and V96P/N142M variant of *E. coli* complex I

The fluorescence of free flavin (the fluorescence of the complex I-bound flavin is quenched by the protein, and is not significant) was monitored using a real-time PCR machine as the temperature was increased. The data points are mean averages ± S.E.M. ($n = 4$). Blue, wild-type, $t_{1/2} = 67.1 \pm 0.1$ °C. Red, V96P/N142M variant, $t_{1/2} = 63.7 \pm 0.1$ °C. Conditions: protein concentration, 1 mg · ml^{-1}, 20 mM Mops buffer, 150 mM NaCl, 10 % glycerol, 0.05 % dodecyl maltose neopentyl glycol, pH 7.5.

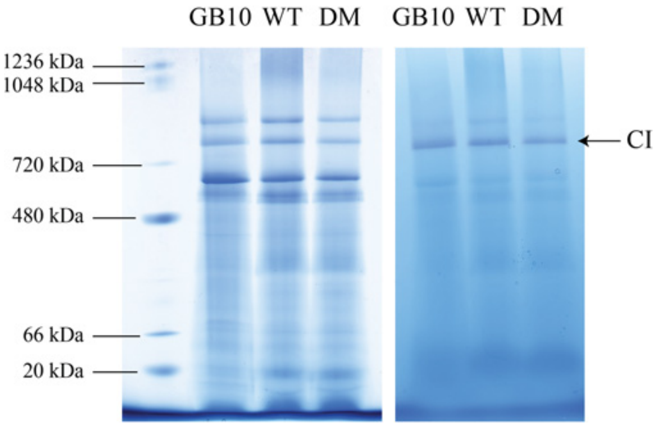

Figure S6 Blue native PAGE analysis of mitochondrial membranes from variants of *Y. lipolytica*

Membranes were solubilized using a detergent/protein ratio of 1.5:1 (protein concentration 10 mg · ml^{-1}, *n*-dodecyl-β-D-maltoside). After centrifugation, 50 μg of solubilized proteins was loaded per lane. The gels were visualised using Coomassie Blue stain (left) or with the complex I in-gel activity assay (right). CI, complex I.

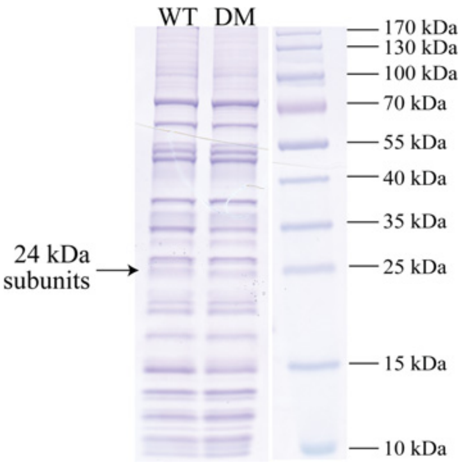

Figure S7 SDS/PAGE analyses of the wild-type and P103V/M149N variants of *Y. lipolytica* complex I

The sizes of a set of molecular-mass markers, and the positions of the 24 kDa subunits, are indicated. WT, wild-type; DM, double mutant (P103V/M149N). A total of 10 μg of protein was loaded per lane, and three lanes have been removed from between the lanes of interest and the marker lane.

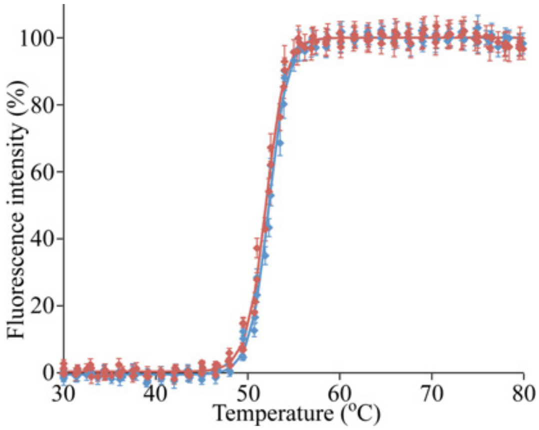

Figure S8 Thermal stability of the flavin site in the WT and P103V/M149N variants of *Y. lipolytica* complex I

The fluorescence of free flavin (the fluorescence of the complex I-bound flavin is quenched by the protein, and is not significant) was monitored using a real-time PCR machine as the temperature was increased. The data points are mean averages ± S.E.M. ($n = 4$). Blue, wild-type, $t_{1/2} = 52.2 \pm 0.1$ °C. Red, P103V/M149N variant, $t_{1/2} = 51.9 \pm 0.1$ °C. Conditions: protein concentration, 2 mg · ml^{-1}, 20 mM Mops buffer, 150 mM NaCl, 10 % glycerol and 0.05 % dodecyl maltose neopentyl glycol, pH 7.5.

© 2013 The Author(s)
The author(s) has paid for this article to be freely available under the terms of the Creative Commons Attribution Licence (CC-BY) (http://creativecommons.org/licenses/by/3.0/) which permits unrestricted use, distribution and reproduction in any medium, provided the original work is properly cited.

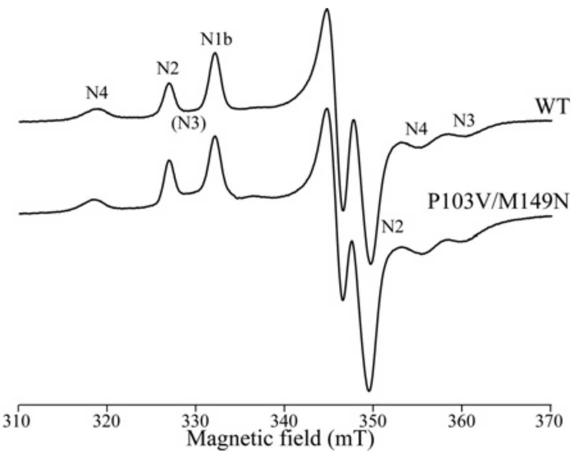

Figure S9 EPR spectra of the [4Fe–4S] clusters in the wild-type and P103V/M149N variant of *Y. lipolytica* complex I

Spectra were recorded at 12 K to focus on the complement of [4Fe–4S] clusters present, using 1 mW microwave power (see the legend to Figure 5 in the main paper for other experimental details). Key features from the clusters observed are marked (the [4Fe–4S] clusters are N2, N3 and N4; the g_z feature of N3 is broad in *Y. lipolytica*, and 'underneath' the signals from N2 and N1b). The spectra of the wild-type (WT) and P103V/M149N variants are essentially indistinguishable.

Table S1 Primers used to generate the mutants and sequence replacements of the 24 kDa subunits in the present study

The primers are named using the subunit to which they were applied (24 kDa for *B. taurus*, nuhm for *Y. lipolytica* and nuoE for *E. coli*) and the mutation/replacement that was made. The mutated positions/replacement sequence are underlined and in italics.

Primer number	Name	Sequence (5′→3′)
1	his-nuhm forward	AGA*CATATG*CATCACCACCATCATCACACCACTCGGGCCCCCCAGGC
2	his-nuhm reverse	TAG*GAATTC*TCATTAAATACCCTCCTGGAGG
3	24 kDa Q101R forward	*G*AGTCTGCACTACCACACCTTGC
4	24 kDa Q101R reverse	GAATGTGATACTTTCCAACAGGC
5	24 kDa D114N forward	*A*ACAGCATACTGGAAGCCATTCAG
6	24 kDa D114N reverse	AGAGTTTCGGAGCATGCAAGGTGTGG
7	24 kDa E143C forward	*TGT*TGTTTAGGGGCCTGTGTAAATGC
8	24 kDa E143C reverse	AACTTCTATAAGAGTGAAAAGTTTGTC
9	24 kDa E143Q forward	*C*AATGTTTAGGGGCCTGTGTAAATGC
10	24 kDa E143Q reverse	AACTTCTATAAGAGTGAAAAGTTTGTC
11	24 kDa N150K forward	*A*AAGCACCAATGGTTCAAATAAATG
12	24 kDa N150K reverse	TACACAGGCCCCTAAACATTCAAC
13	24 kDa Δloop1 forward	TCTGACAGCATACTGGAA
14	24 kDa Δloop1 reverse	GACTTGAATGTGATACTT
15	nuoE loop1 forward	*TGTGACAGCGTGGTCTGTCATATCAACGGT*
16	nuoE loop1 reverse	*ACCGTTGATATGACAGACCACGCTGTCACA*
17	nuoE 24kDa loop1 forward	*ATGCTCCGAAAC*TATCAGGGTATTCAGGCGGC
18	nuoE 24kDa loop1 reverse	*GCAAGGTGTGGTAGT*GCAATAACGGATCACATGG
19	24 kDa Δloop2 forward	GCACCAATGGTTCAAATA
20	24 kDa Δloop2 reverse	TTCTATAAGAGTGAAAAG
21	nuoE loop2 forward	*ACTTGCTGCCTGGGGAACTGTGATAAA*
22	nuoE loop2 reverse	*TTTATCACAGTTCCCCAGGCAGCAAGT*
23	nuhm nuoE loop1 forward	*GTCACATCAAT*GGCTCCGACGGTATCATGGAGG
24	nuhm nuoE loop1 reverse	*AGACGACGCTGTC*ACAGATCTGCAGATGGTACC
25	24 kDa nuoE CT-β forward	GACAACTACTATGAGG
26	24 kDa nuoE CT-β reverse	*ATCGATCATCATGTTTGGCCC*ATTTACACAGGCCCCTAAAC
26	24 kDa M153N forward	A*AC*GTTCAAATAAATGAC
28	24 kDa M153N reverse	TGGTGCATTTACACA
29	nuhm M149N forward	A*AC*ATGGCCATCAACGACG
30	nuhm M149N reverse	GGGGGCGTTGACACAGGC
31	nuoE N142M forward	A*TG*ATGATGATCGATGAGGAC
32	nuoE N142M reverse	TGGCCCTTTATCACAGTTCCC
33	nuhm P103V forward	*GT*CTGTCAGCTGTGTGGCTCCG
34	nuhm P103V reverse	GGTGGTGGTACAGATCTGC
35	nuoE V96P forward	*CCG*TGTCATATCAACGGTTATCAGG
36	nuoE V96P reverse	CCTGATAACCGTTGATATGACA

© 2013 The Author(s)
The author(s) has paid for this article to be freely available under the terms of the Creative Commons Attribution Licence (CC-BY) (http://creativecommons.org/licenses/by/3.0/) which permits unrestricted use, distribution and reproduction in any medium, provided the original work is properly cited.

Table S2 Primers used to generate mutations in intact complex I from *Y. lipolytica* and *E. coli*

The primers are named using the subunit to which they were applied (nuhm for *Y. lipolytica* and nuoE for *E. coli*) and the mutation that was made. The mutated positions are underlined and in italics. In addition, a T/A to C/G change was incorporated in the nuoE V96P 2 forward and reverse primers to prevent them forming a hairpin, two C/G to T/A changes were made in the nuoE V96P 2 forward and reverse primers to introduce a VspI restriction site, and an A/T to C/G change was made in the N142M 2 forward and reverse primers to introduce a BanII restriction site.

Primer number	Name	Sequence (5′→3′)
29	nuhm M149N forward	A*AC*ATGGCCATCAACGACG
30	nuhm M149N reverse	GGGGGCGTTGACACAGGC
33	nuhm P103V forward	*GT*CTGTCAGCTGTGTGGCTCCG
34	nuhm P103V reverse	GGTGGTGGTACAGATCTGC
37	nuoE V96P 2 forward	GTGACAGCGTG*CCG*TG*C*CATAT*T*AA*T*GGTTATCAGG
38	nuoE V96P 2 reverse	CCTGATAACC*A*TT*A*ATATG*G*CA*CGG*CACGCTGTCAC
39	nuoE N142M 2 forward	GTGATAAAGGGCC*C*A*TG*ATGATGATCGATG
40	nuoE N142M 2 reverse	CATCGATCATCAT*CA*T*G*GGCCCTTTATCAC
41	nuoE linear forward	CACGAGAATCAACAACCACAAACCG
42	nuoE linear reverse	TTTATACCGCTCCAGCAGTTCAGG

Table S3 Catalytic activity measurements in *E. coli* membranes containing variant forms of complex I

The measurements were made using membranes prepared from an *E. coli* strain in which the chromosomal genes for the alternative NADH dehydrogenases have been removed by λ-red-mediated recombination (D. Dekovic and T. Friedrich, unpublished work), so the values are specific to only complex I. NADH:FeCN oxidoreduction is catalysed by the complex I flavin site and was performed in the presence of piericidin A to inhibit quinone reduction. NADH:O_2 oxidoreduction (>99 % sensitive to piericidin A) refers to catalysis by the whole respiratory chain. Measurements were conducted at 30 °C in 50 mM Mes/OH (pH 6.0), 50 mM NaCl and 5 mM $MgCl_2$, using 200 μM NADH and 1 mM ferricyanide, or 1 mM NADH for the NADH:O_2 oxidoreduction measurements.

	NADH:FeCN oxidoreduction		NADH:O_2 oxidoreduction	
E. coli complex I variant	μmol · min^{-1} · mg^{-1}	%	μmol · min^{-1} · mg^{-1}	%
Wild-type	1.6 ± 0.1	(100)	0.25 ± 0.02	(100)
V96P	1.3 ± 0.1	81 ± 8	0.21 ± 0.02	84 ± 8
V96P/N142M	1.2 ± 0.1	75 ± 8	0.20 ± 0.02	80 ± 10

Table S4 Catalytic activity measurements on the flavin sites of variant forms of *Y. lipolytica* complex I

See the legend to Figure 6 in the main paper for the assay conditions.

	NADH:FeCN oxidoreduction		NADH:APAD$^+$ oxidoreduction		NADH:O_2 oxidoreduction (H_2O_2 production)	
Y. lipolytica complex I variant	μmol · min^{-1} · mg^{-1}	%	μmol · min^{-1} · mg^{-1}	%	nmol · min^{-1} · mg^{-1}	%
Wild-type	116 ± 1	(100)	4.9 ± 0.1	(100)	27 ± 1	(100)
P103V	98 ± 1	84 ± 1	4.5 ± 0.1	92 ± 3	25 ± 1	93 ± 5
M149N	58 ± 5	50 ± 4	2.7 ± 0.1	55 ± 2	16 ± 1	59 ± 4
P103V/M149N	52 ± 2	45 ± 2	2.8 ± 0.1	57 ± 2	9.5 ± 0.5	35 ± 2

Received 30 April 2013/14 August 2013; accepted 27 August 2013
Published as BJ Immediate Publication 27 August 2013, doi:10.1042/BJ20130606

© 2013 The Author(s)
The author(s) has paid for this article to be freely available under the terms of the Creative Commons Attribution Licence (CC-BY) (http://creativecommons.org/licenses/by/3.0/) which permits unrestricted use, distribution and reproduction in any medium, provided the original work is properly cited.
